# Adaptive reliance on the most stable sensory predictions enhances perceptual feature extraction of moving stimuli

**DOI:** 10.1152/jn.00850.2015

**Published:** 2016-01-28

**Authors:** Neeraj Kumar, Pratik K. Mutha

**Affiliations:** ^1^Centre for Cognitive Science, Indian Institute of Technology Gandhinagar, Ahmedabad, Gujarat, India; and; ^2^Department of Biological Engineering, Indian Institute of Technology Gandhinagar, Ahmedabad, Gujarat, India

**Keywords:** forward model, motor control, motor learning, perception, sensory predictions

## Abstract

The prediction of the sensory outcomes of action is thought to be useful for distinguishing self- vs. externally generated sensations, correcting movements when sensory feedback is delayed, and learning predictive models for motor behavior. Here, we show that aspects of another fundamental function—perception—are enhanced when they entail the contribution of predicted sensory outcomes and that this enhancement relies on the adaptive use of the most stable predictions available. We combined a motor-learning paradigm that imposes new sensory predictions with a dynamic visual search task to first show that perceptual feature extraction of a moving stimulus is poorer when it is based on sensory feedback that is misaligned with those predictions. This was possible because our novel experimental design allowed us to override the “natural” sensory predictions present when any action is performed and separately examine the influence of these two sources on perceptual feature extraction. We then show that if the new predictions induced via motor learning are unreliable, rather than just relying on sensory information for perceptual judgments, as is conventionally thought, then subjects adaptively transition to using other stable sensory predictions to maintain greater accuracy in their perceptual judgments. Finally, we show that when sensory predictions are not modified at all, these judgments are sharper when subjects combine their natural predictions with sensory feedback. Collectively, our results highlight the crucial contribution of sensory predictions to perception and also suggest that the brain intelligently integrates the most stable predictions available with sensory information to maintain high fidelity in perceptual decisions.

it is widely believed that when the brain generates motor commands to produce an action, it also predicts the sensory feedback that might be expected as a consequence of that action (Bar 2007; Bubic et al. 2010; Hommel et al. 2001; Synofzik et al. 2006; von Holst and Mittelstaedt 1950; Waszak et al. 2012). Such predicted action outcomes rely on prior knowledge of the properties of the body and the environment and are thought to be the output of an internal “forward model,” which uses a copy of the outgoing motor commands as its input (Wolpert and Miall 1996). Predicted action outcomes have been hypothesized to carry immense benefits for coordinated behavior (Miall et al. 2007; Schiffer et al. 2015; Shadmehr et al. 2010; Wolpert and Flanagan 2001). Fundamental among these is the notion that these signals are a source of rich sensory information that can be used in conjunction with actual sensory feedback to yield better perceptual estimates of the state of the body and the world. In other words, just as the integration of inputs from multiple sensory modalities, or multisensory integration, is thought to give rise to better perception (DeAngelis and Angelaki 2012; Ernst and Banks 2002; van Beers et al. 1999), it is postulated that the integration of predicted sensory outcomes with actual sensory feedback can yield perceptual estimates that are sharper than those possible by relying on either source alone. One plausible underpinning for this is that better perception arises because predicted action effects may reach the threshold of awareness faster, giving rise to a more detailed stimulus representation (Kok et al. 2012, 2013). Nevertheless, compelling experimental evidence directly supporting this idea of enhanced perceptual estimates is unfortunately scarce.

A convincing case substantiating this notion can be made if it can be demonstrated that decisions about perceptual features extracted from sensory observations alone are less accurate than those derived by integrating predicted action outcomes and sensory feedback. However, when an action is made, the isolation of perceptual estimates derived solely from sensory feedback is challenging, because motor commands associated with that action will always yield sensory predictions (through the operation of the forward model), which can then contribute to those estimates (Girshick et al. 2011). Furthermore, for the same action, these “default” or natural action outcome predictions can vary across individuals depending on their prior knowledge and/or individual beliefs about the body and the environment, which can then lead to differences in perceptual decisions (Kording and Wolpert 2004). In such cases, what may appear to be a suboptimal decision may actually be optimal from an individual standpoint. Here, we sought to overcome these challenges and first assess whether decisions about perceptual attributes are indeed better when action outcome predictions and sensory feedback are aligned and can be integrated and conversely, worse when they are based on sensory feedback alone.

To do so, we combined a motor-learning paradigm with a visual perceptual task. The motor-learning task was critical, since it allowed us to artificially impose similar, stable sensory predictions across subjects (Synofzik et al. 2006), thereby over-riding their natural predictions, which as stated above, could theoretically result in very different perceptual estimates across individuals. Motor learning was achieved via a visuomotor adaptation paradigm, in which subjects learned to adapt their movements to distorted visual feedback of their hand motion. Specifically, this visual feedback was rotated by 10° relative to actual hand motion. Adaptation to this rotation resulted in an update of subjects' sensory predictions. In the perceptual task, subjects searched for and reported the color of a predefined visual target that moved on a screen among other moving distractors while they also moved their unseen hand. Crucially, target motion in this dynamic visual search task was such that it was either consistent or inconsistent with the subjects' predictions that had been updated via visuomotor adaptation. In other words, in this task, the target moved randomly, congruent with the actual hand motion (hereafter referred to as “hand aligned”) or along a direction that was rotated by 10° relative to hand motion (hereafter referred to as “rotated”). We reasoned that when visual feedback of target motion was rotated and therefore, consistent with the subjects' modified predictions, these two streams of information would be combined to yield a more accurate perceptual decision about target color. Conversely, if motion of the target was hand aligned and therefore, inconsistent with their modified predictions, then subjects would rely on visual feedback alone for their perceptual judgments, and the accuracy of the color report would be poorer. We tested this in our first experiment.

In our second experiment, we asked how the accuracy of perceptual feature extraction would be affected if the modified action outcome predictions were unreliable. Specifically, we examined whether subjects would continue to rely on their unreliable predictions, use sensory information alone, or transition to using different, more reliable predictions when making perceptual judgments. We made the novel prediction that in this case, subjects would adaptively switch to relying on their natural sensory predictions, available when performing any action, so that greater fidelity in their perceptual judgments is maintained. Finally, based on the findings of our first two experiments, we posited that if the predictions were not modified artificially at all, then subjects would simply rely on their natural predictions to make perceptual judgments, which would also be reflected in the accuracy of their color report. We tested this in our final experiment.

## METHODS

### Subjects

A total of 36 young, healthy, right-handed individuals with normal or corrected-to-normal vision participated in the study. No subject reported any history of neurological disorders or orthopedic injuries. All subjects provided informed consent before participation. The study was approved by the Institute Ethics Committee of the Indian Institute of Technology Gandhinagar.

### Setup

**Fig. 1.**
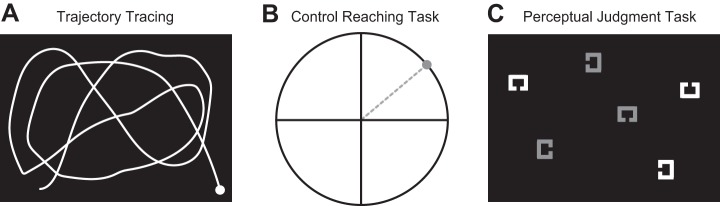
Experimental tasks. *A*: example trajectory to be traced during the visuomotor adaptation task. Subjects were asked to trace this trajectory (with the white circle as the start position) on a digitizing tablet using a hand-held stylus. Visual feedback was given by the means of a cursor. *B*: control reaching task. Subjects had to make a pointing movement (gray, dashed line) toward a given target (gray circle). No visual feedback was given. *C*: perceptual judgment task (modified visual search). Subjects were required to search for and identify the color [red (shown in *C* as gray) or green (shown in *C* as white)] of a target stimulus, defined before each trial by the position of the gap in its outline. In the example, the target is defined as the square with a gap on the right side.

The experimental setup was composed of a pseudo-virtual reality system, in which subjects sat facing a horizontally mounted computer screen that was positioned above a digitizing tablet. Subjects made planar movements by moving a hand-held stylus on the tablet. Direct visual feedback of the hand was not available, since it was blocked by the computer screen. Instead, visual feedback about stylus position (and thereby, hand position) was given by means of an on-screen cursor. The position of the cursor could either be veridical or distorted relative to hand motion. Start positions and randomly curved trajectories for tracing (for *experiments 1* and *2*) were also displayed on the computer screen (Fig. 1*A*). The tracing task was not used in *experiment 3*. The same setup was used for the perceptual judgment task.

### Visuomotor Adaptation Task

Subjects were asked to trace a displayed trajectory from a defined start position located at one of its ends (Fig. 1*A*). For each trial, the trajectory to be traced was randomly selected from a set of 30 curved trajectories that had been previously generated by the experimenter. This trajectory was shown for 20 s/trial. If subjects finished tracing the full path within 20 s, then the trajectory was automatically elongated. Thus subjects had to move for 20 s on each trial. Subjects received visual feedback about their hand position by means of the cursor, but cursor motion was rotated by 10° in the clockwise direction relative to hand motion. The magnitude of the cursor rotation remained fixed at 10° for every trial. Angular error between the hand and cursor positions was calculated every 1 s, and its mean value for each path was derived online. This direction error was computed as the angle between two lines: the line connecting the previous and the current locations of the cursor and the line connecting the corresponding perpendicular points on the displayed trajectory. Clockwise errors were considered positive. Subjects continued the trajectory-tracing task until the mean error between hand and cursor motion was smaller than 0.5°, averaged over the entire trial.

### Control Reaching Task

Upon exposure to the 10° rotation, subjects began modifying the direction of their hand motion and over a few trials, adapted to the rotation. To confirm that adaptation indeed occurred and that the subjects' predictions had been updated, we used a control task in which subjects were required to perform a simple point-to-point reaching movement. A white circle (diameter = 10°) was presented on the computer screen with a small circular start position located at its center (Fig. 1*B*). A red pointing target (filled circular disc; diameter = 0.5°, shown in Fig. 1*B* as the gray circle) was presented in one of four different positions in the first quadrant of the larger white circle (at the 12-, 1-, 2-, or 3-o'clock positions). Upon receiving a go signal, subjects were asked to perform an out-and-back pointing movement from the start circle to the target. No visual feedback was provided on these trials. For these trials, we calculated movement error as the angle between the line connecting the start position to the reversal point of the actual movement and the line connecting the start position to the target.

### Perceptual Judgment Task

Subjects also performed a dynamic equivalent of a visual search task modeled after Salomon et al. (2011). Square stimuli were displayed against a black background, inside an invisible black rectangle subtending 89 × 112 mm, centered at the middle of the computer monitor (Fig. 1*C*). In each search display, six outlined squares were presented. Each square had sides measuring 6.4 mm with a 1-mm gap on any one of the four sides. Before each search trial, a single white square, similar to the squares appearing during the search trial, was shown at the center of the screen. This served as the target for the upcoming search trial. The remaining squares served as distractors. For the target and one other square, the side containing the 1-mm gap was unique. However, for each set of two of the remaining four squares in the search display, the side containing the 1-mm gap was identical. Each square was either light red (RGB = 145,140,125) or light green (RGB = 125,145,131). These values yielded colors that could not be distinguished easily. Each search display consisted of an equal number of green and red items. Subjects were asked to report the color of the target (red or green) during the search trial by pressing designated keys (“Ctrl” for red and “Alt” for green, counterbalanced across subjects) with their left hand as quickly and accurately as possible.

While performing this search task, subjects also moved the stylus with their right hand on the digitizing tablet throughout the trial. They were instructed that these right-arm movements must be continuous, cover all four quadrants of the display, be neither too fast nor too slow, and start with the presentation of the “start moving” instruction. The motion of one of the stimulus squares of the search display, which could be the target, followed the motion of the subject's hand in real time, whereas the motion of the other stimuli (distractors) followed a path that was a distorted version of the motion of the hand. This distorted path was generated by rotating the hand motion by a random value between 30° and 70°. On any given trial, this value was different for each stimulus. Although subjects were informed that their hand controlled the movement of one of the stimuli, they were also told that this item was not more likely than another to be the target, and so they should not try to look for it. To avoid the situation that the self-controlled stimulus was stationary and therefore, obvious among the rest of the moving stimuli at trial onset, each trial began with a start-moving written instruction that instructed the subjects to begin their movements (without any visual feedback) before the onset of the search display. After 1,200 ms, the search display appeared with all stimuli, including the self-controlled stimulus already in motion. Since the motion of the stimuli was either directly congruent with the hand or some rotationally transformed version of it, all stimuli moved at the same speed as the hand. The search display was presented until subjects made a response or until 6 s had elapsed. An individual square did not rotate during motion. Subjects performed a total of 192 search trials. The accuracy of the color report was recorded for further analysis.

### Tone-Discrimination Task

In *experiment 2*, subjects performed a tone-discrimination task as they adapted to the visuomotor rotation. This additional task was used to create fragility in the adaptation-induced update to the subjects' sensory predictions (Taylor and Thoroughman 2007). For this, subjects were first screened for their ability to discriminate the frequency of two tones. They performed 100 two-interval, two-alternative forced-choice frequency discriminations. The first tone was centered at 2,000 ± 100 Hz, whereas the frequency of the second tone was changed randomly between ±1 and ±150 Hz relative to the first tone. Both tones were presented for 100 ms at identical volumes with a gap that ranged from 150 to 450 ms. Subjects were instructed to determine quickly and accurately whether the second tone was of higher or lower pitch than the first tone. Subjects made their discrimination decisions by pressing one of two buttons (Ctrl or Alt) with their left hand that corresponded to higher or lower frequency. Each frequency change was repeated 10 times. After each discrimination, subjects were provided with feedback about the accuracy of their response. All tones were encoded to 16 bits at 16 kHz and played through headphones. Subjects were allowed to adjust the volume of the headphones to suit their comfort. Accuracy was recorded for each tone.

Immediately after the screening, subjects performed the frequency discrimination task without the adaptation task. In this task, the first tone was again centered at 2,000 ± 100 Hz. The specific change in frequency for the second tone was determined from each subject's performance on the prior screening task. The tone was such that it had resulted in 85–95% correct discriminations during screening. The time between two successive tones was set to 200 ms. These same conditions were integrated with the adaptation task to create the dual-task condition in *experiment 2*. Accuracy of correct discriminations was determined, and it was noted that accuracy of tone discrimination decreased during the dual-task (tone discrimination plus adaptation) condition compared with when discrimination was tested alone.

### Task Progression

#### Experiment 1.

In *experiment 1*, 12 subjects (mean age = 21.6 ± 0.5 yr; 8 men) first adapted to the 10° visuomotor rotation and then performed the visual search task. During the perceptual task, the square target, whose color had to be identified, moved in one of three different ways on any given trial: hand aligned, rotated 10° relative to the hand and random. Random target motion was generated by rotating the actual hand motion by a value drawn randomly from 30° to 70°. The target was equally likely to follow all three motion conditions. Subjects also performed the control-reaching task before and after the motor adaptation task to confirm that adaptation had indeed occurred.

#### Experiment 2.

In *experiment 2*, 12 subjects (mean age = 21.8 ± 0.5 yr; 10 men) performed the same adaptation task, where they were required to adapt to the 10° cursor rotation. However, during adaptation, they also performed the tone-discrimination task. Following this dual task, subjects performed the dynamic visual search task. The conditions for this task remained the same *as experiment 1*. In addition, subjects performed the control-reaching task before and after the adaptation task.

#### Experiment 3.

In *experiment 3*, 12 subjects (mean age = 22.4 ± 0.5 yr; 9 men) performed only the search task without the adaptation or the control reaching task. In the search task, only two target motion conditions were imposed: hand aligned and random.

## RESULTS

### Experiment 1

**Fig. 2.**
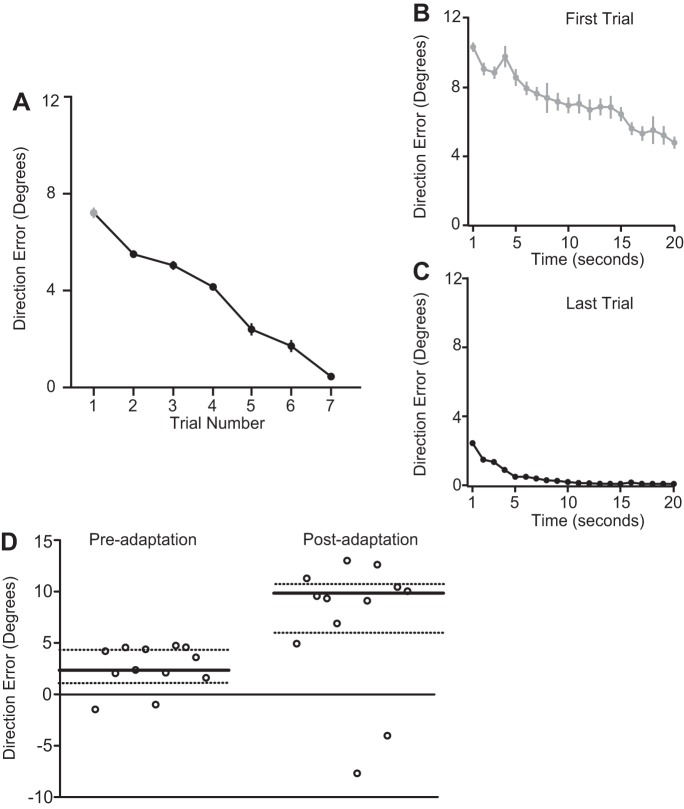
Visuomotor adaptation during trajectory tracing in *experiment 1*. *A*: reduction in direction error across trials. *B*: reduction in direction error during the first 20-s tracing trial. *C*: reduction in direction error during the last 20-s tracing trial. *D*: mean direction error for each subject during the control reaching task in the pre- and postadaptation phases. Median values (bold lines) and quartiles (dotted lines) are shown. Error bars represent SE.

In our first experiment, subjects initially adapted to a 10° visuomotor rotation while they traced with their unseen hand a randomly curved trajectory displayed on a screen for a number of trials, each lasting 20 s. When the rotation was first introduced, subjects showed errors in movement direction, which were almost equal to the magnitude of the rotation (mean error on the first second of the first learning trial: 10.3 ± 0.30°; Fig. 2*B*). However, over the course of the 20-s trial, these direction errors decreased (mean error on the 20th second of the first learning trial: 4.8 ± 0.40°; Fig. 2*B*). This learning was sustained across trials, such that mean error on subsequent trials was smaller than that on the previous trial (Fig. 2*A*). Mean error on the first second of the last learning trial was 2.4 ± 0.2°, whereas on the 20th second, it had reduced to 0.06 ± 0.006° (Fig. 2*C*). Thus over a few trials (mean ± SE = 6.58 ± 0.66 trials), the rotation was almost completely compensated, and direction error was close to zero, a significant change from the error observed on the first trial [t(11) = 37.68, *P* < 0.001, Fig. 2*A*]. Adaptation was confirmed by the presence of after-effects in the control reaching task (Fig. 2*D*) in which subjects made out-and-back reaching movements to four targets. In this task, whereas subjects made pointing errors of 2.97 ± 2.13° before adaptation, they showed clear after-effects and direction errors of 9.43 ± 6.50° after adapting to the 10° rotation [t(11) = 2.66, *P* = 0.02]. Collectively, the reduction in error during adaptation and the presence of after-effects in the control task are indicators of updates to internal predictions about sensory action outcomes (Krakauer 2009; Mutha et al. 2011; Synofzik et al. 2006).

**Fig. 3.**
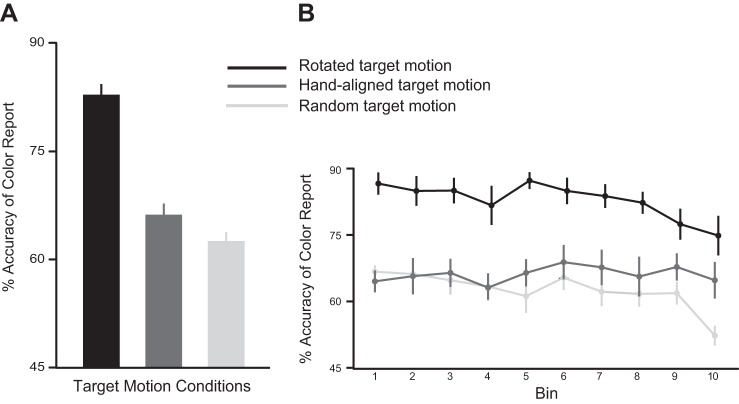
Performance during the visual search task of *experiment 1*. *A*: mean percent accuracy of the color report for the hand-aligned (dark gray), rotated (black), and random (light gray) target motion conditions. *B*: mean percent accuracy divided into bins. Accuracy was always highest in the condition where the target moved along the rotated path. Error bars represent SE.

After adaptation, subjects performed the search task in which the target could move in a random, hand aligned, or 10° rotated direction. The reaction time (RT) for reporting the color of target was similar in all three conditions [mean RT for rotated motion = 3.65 ± 0.15 s, hand-aligned motion = 3.72 ± 0.12 s, and random motion = 3.67 ± 0.12 s; one-way ANOVA F(2,22) = 0.10, *P* = 0.90]. However, as shown in Fig. 3*A*, the accuracy of the color report was greatest when the target moved along the rotated path compared with random or hand-aligned motion [mean accuracy for rotated motion = 82.75 ± 1.60%, hand-aligned motion = 66.08 ± 1.68%, and random motion = 62.41 ± 1.38%; one-way ANOVA F(2,22) = 71.76, *P* < 0.001]. We explored whether this accuracy showed any time-dependent trends by dividing the search trials into bins, each containing 10% of the trials (Fig. 3*B*). We observed a significant bin (first, last) X target motion (random, hand aligned, rotated) interaction [two-way ANOVA F(2,55) = 5.31, *P* = 0.007]. Importantly, post hoc Tukey's tests revealed that accuracy was significantly higher (*P* < 0.001) when the target moved along the rotated path compared with the other target motion conditions during the first bin (rotated vs. random: *P* < 0.001; rotated vs. hand aligned: *P* < 0.001), as well as the last bin (rotated vs. random: *P* < 0.001; rotated vs. hand aligned: *P* = 0.04). Mean accuracy of the color report for the target moving along the rotated path was 86.50 ± 2.29% in the first bin, whereas it was 66.58 ± 1.20% and 64.41 ± 2.24% for targets moving in the random and hand-aligned directions, respectively. This difference was maintained even on the last bin of search trials, where mean accuracy for targets moving along the rotated path was 74.75 ± 4.24% compared with 64.66 ± 3.90% and 52.16 ± 2.03% in the hand-aligned and random motion conditions, respectively. The decline in accuracy from the first to the last bin was significant in the rotated (*P* = 0.01) and random (*P* = 0.001) conditions but not the hand-aligned direction (*P* = 0.99). Nonetheless, throughout the search task, accuracy of the perceptual decision was greatest if the target moved not with the hand but along the rotated path, i.e., when actual (feedback about) target motion was consistent with the subjects' newly imposed stable predictions.

### Experiment 2

**Fig. 4.**
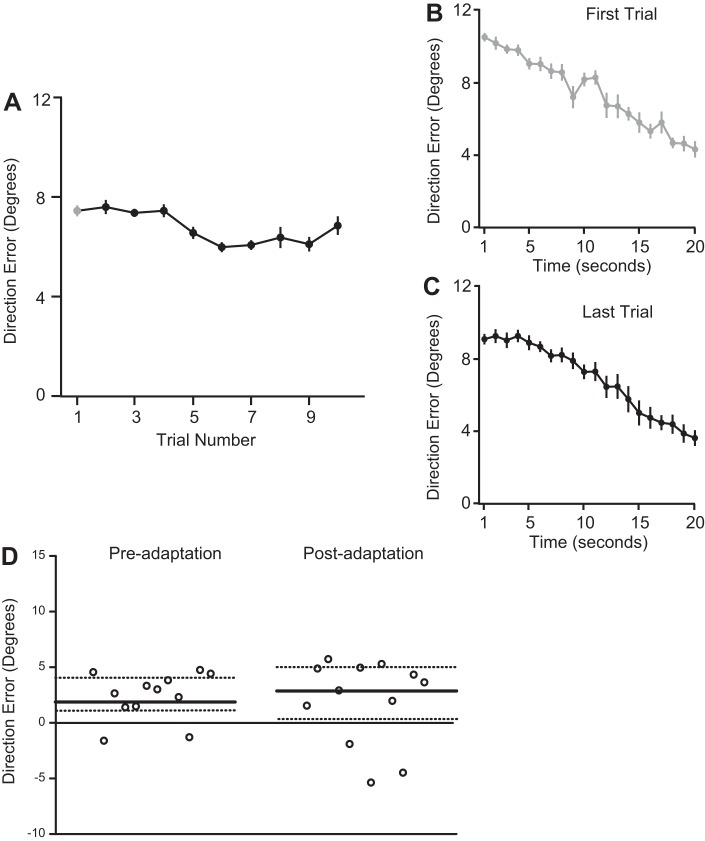
Lack of adaptation during trajectory tracing task in *experiment 2*. *A*: mean direction error across trials. *B*: reduction in direction error during the first 20-s tracing trial. *C*: reduction in direction error during the last 20-s tracing trial. The first and last trials appear the same. *D*: mean direction error for each subject in the control reaching task during the pre- and postadaptation phases. Median values (bold lines) and quartiles (dotted lines) are shown. Error bars represent SE.

In our second experiment, subjects were required to perform a tone-discrimination task simultaneously with the adaptation task. This tone-discrimination task did not prevent reduction in errors during the 20-s trajectory tracing trial. Mean error on the first second of the first trial was 10.51 ± 0.24°, which reduced to 4.29 ± 0.47° by the 20th second (Fig. 4*B*), a reduction that was clearly statistically significant [t(11) = 12.83, *P* < 0.001]. This pattern was also noted on the 10th adaptation trial [mean error on first second = 9.08 ± 0.30°, mean error on 20th second = 3.60 ± 0.45°, significant decrease from 1st to 20th second, t(11) = 13.26, *P* < 0.001, Fig. 4*C*]. However, the within-trial improvement was not sustained across trials. Mean error on the 10th trial of the adaptation block was not significantly different than that of the first trial [mean error on first trial = 7.5 ± 0.60°; last trial = 6.88 ± 0.57°, t(11) = 1.36, *P* = 0.20; Fig. 4*A*]. Since subjects failed to show an improvement across the 10 trials (as opposed to ∼7 trials in *experiment 1*), the adaptation session was stopped. The lack of adaptation was confirmed in the targeted reaching control task (Fig. 4*D*). Mean preadaptation direction error in the control task was 2.82 ± 2.11°, whereas the postadaptation direction error was 3.27 ± 3.84°. The lack of significant difference in pre- and postadaptation errors [t(11) = 0.40, *P* = 0.69] pointed to a clear lack of after-effects, confirming that subjects did not adapt to the rotation. Thus the tone-discrimination task prevented the formation of a stable memory of the rotation, thereby preventing a sustained update of the subject's sensory predictions.

**Fig. 5.**
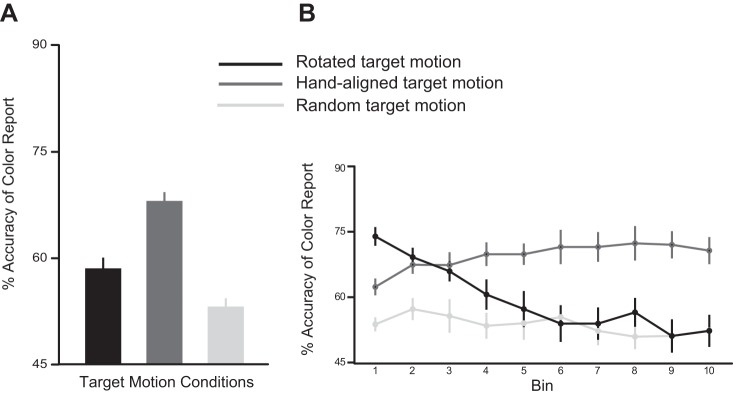
Performance during the visual search task of *experiment 2*. *A*: mean percent accuracy of the color report for the hand-aligned (dark gray), rotated (black), and random (light gray) target motion conditions. *B*: mean percent accuracy divided into bins. Accuracy was greater initially for the rotated targets. However, with time, accuracy became greater for the hand-aligned targets. Error bars represent SE.

In the dynamic visual search task that followed the adaptation task, RT for reporting color of target was again similar in all three conditions [mean RT for rotated motion = 3.73 ± 0.09 s, hand-aligned motion = 3.80 ± 0.08 s, and random motion = 3.98 ± 0.14 s; one-way ANOVA F(2,22) = 1.11, *P* = 0.34]. Overall, accuracy of the color report was greater for the target that moved in the hand-aligned direction rather than along the rotated path [Fig. 5*A*; one-way ANOVA, F(2,22) = 32.66, *P* < 0.001]. Interestingly, however, when we split the search trials into bins containing 10% of the trials, a significant bin (first, last) X target motion (random, hand aligned, rotated) interaction [two-way ANOVA, F(2,55) = 20.32, *P* < 0.001; Fig. 5*B*] was observed. Post hoc Tukey's tests revealed that accuracy of the color report was significantly higher for the rotated target compared with the other target motion conditions in the first bin (mean accuracy for rotated condition = 73.75 ± 1.91%, hand aligned condition = 62.16 ± 1.69%, random condition = 53.58 ± 1.37%; rotated vs. random: *P* < 0.001, rotated vs. hand aligned: *P* = 0.01). However, in the last bin, accuracy was greatest for targets moving in the hand aligned direction and not along the rotated direction (mean accuracy for rotated condition = 52.08 ± 3.43%, hand aligned condition = 70.5 ± 2.87%, random condition = 52.16 ± 2.02%; rotated vs. random: *P* = 0.99, rotated vs. hand aligned: *P* < 0.001). This suggested that subjects initially relied on their modified predictions, but since these predictions were short lived and therefore, unreliable, rather than just depending on sensory information alone, subjects dynamically transitioned to using other, more stable predictions to maintain greater accuracy in their perceptual decisions.

### Experiment 3

**Fig. 6.**
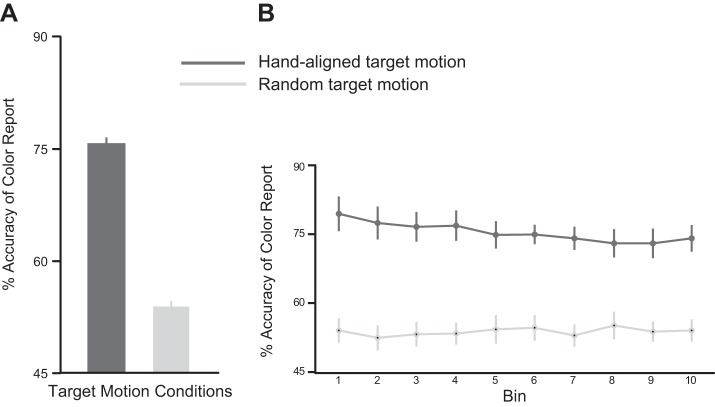
Performance during the perceptual task of *experiment 3*. *A*: mean percent accuracy of the color report for the hand-aligned (dark gray) and random (light gray) target motion conditions. *B*: mean percent accuracy divided into bins, with each bin containing 10% of the total trials. Accuracy was always greater for the hand-aligned targets. Error bars represent SE.

We wondered whether the pattern of results seen in *experiment 2* emerged because subjects actually switch from initially relying on their weakly updated predictions to later, using their default, natural predictions. If so, then we predicted that in a “baseline” perceptual task, subjects should be more accurate in reporting the color of targets moving in the hand-aligned direction than in a random direction, for which perceptual information must be derived based on sensory information alone. We tested this idea in *experiment 3* and found this to be the case. Accuracy of the color report was significantly greater [t(11) = 8.70, *P* < 0.001] for the hand-aligned targets than the randomly moving targets (Fig. 6*A*). This was the case throughout the search task (Fig. 6*B*). Our bin (first, last) X target motion (hand aligned, random) motion did not reveal a significant interaction [F(1, 33) = 1.51, *P* = 0.23] but only a significant main effect of target motion [F(1, 33) = 109.23, *P* < 0.001]. Thus perceptual accuracy was indeed better when subjects could integrate their natural predictions with sensory feedback, and it suffered when these two sources were misaligned.

In summary, across the three experiments, we noted that accuracy of perceptual judgments is enhanced when sensory predictions are integrated with actual sensory feedback. We also newly discovered that to maintain greater accuracy in the ability to extract perceptual information, subjects adaptively switch to relying on the most stable predictions available.

## DISCUSSION

For the current work, we used as a starting point the key suggestion made across a number of studies—that motor commands used to generate an action are also used to predict the sensory outcomes that might arise as a consequence of that action (Bar 2007; Bubic et al. 2010; Hommel et al. 2001; Synofzik et al. 2006; von Holst and Mittelstaedt 1950; Waszak et al. 2012). We demonstrate that use of these predicted action outcomes results in superior extraction of perceptual features of moving stimuli and that this enhancement relies on the adaptive use of the most stable predictions available. In this demonstration, two novel aspects of our study stand out: first, we show that perceptual decisions are poorer if they are based just on sensory observations. During active motion, this is nontrivial, because default action outcome predictions, available when any action is made, can always contribute, along with sensory feedback, during perceptual decisionmaking. We over-rode the influence of these predictions by imposing new, stable predictions via visuomotor adaptation and were then able to force perceptual estimates on sensory feedback alone by making the motion of the target in the perceptual task misaligned with the subjects' sensory expectations. In this case, the accuracy of the perceptual judgment was clearly worse compared to when sensory feedback and predictions were aligned. Second, we show, perhaps for the first time, that when some sensory predictions are unreliable, subjects dynamically switch to using whatever other stable predictions might be available for perceptual judgments. In our case, these were likely the subjects' natural predictions. This finding reveals the tremendous degree of sophistication within the nervous system, where in case of unstable predictions, perceptual estimates and decisions are not derived simply based on sensory information, as is typically suggested (Kording and Wolpert 2004), but rather, the most stable predictions available are co-opted to maintain higher fidelity of perceptual estimates and decisions. Finally, we also show that when the subjects' predictions are not modified, perceptual judgments are sharper when they entail the use of the subjects' natural predictions, along with sensory feedback, compared with conditions where these two sources of information are misaligned.

One of the ways in which a misalignment between the subjects' predictions and target motion was created in the perceptual judgment task was using a condition in which the target moved in a “random” direction relative to the hand (see methods). In this condition, however, one of the distractors moved congruent with the hand. Therefore, it could be argued that the accuracy of the perceptual judgment in the random condition was poor, because motion of a hand (and therefore, prediction)-aligned distractor was more distracting than other conditions, thereby worsening performance. However, our data suggest that this is unlikely to be the case. In *experiment 1*, the accuracy of the perceptual judgment in the random condition was not different than that in the hand-aligned condition in which target motion was congruent with the hand but inconsistent with the subjects' predictions (*P* = 0.13). Furthermore, in *experiment 2*, accuracy in the random condition was also not different than the rotated condition after the subjects had transitioned to using their stable, natural predictions for perceptual judgments (approximately the last 6 bins in Fig. 5*B*); during this time again, target motion was inconsistent with the subjects' predictions. This implies that for judging and reporting the color of the target, all conditions in which target motion was inconsistent with the subjects' predictions were identical. Thus a condition in which a distractor moved consistent with the subjects' predictions (and therefore, the target did not) was not any more distracting than other conditions in which target motion was inconsistent with the subjects' predictions. If this were the case, accuracy in the random condition would have been much worse than the other conditions. Thus whereas we did not include a condition in which the motion of all stimuli in the perceptual task was truly random (there was always one stimulus that moved with the hand), this is unlikely to have impacted our results.

### Influence of Sensory Predictions on Perceptual Estimates

Prior work, examining the role of predicted sensory action outcomes in coordinated behavior, has largely focused on the suggestion that such predictions are helpful in distinguishing whether incoming sensory information is the result of one's own action or due to changes in the environment (Blakemore et al. 1999; Haarmeier et al. 2001; Sperry 1950; Synofzik et al. 2006; von Holst and Mittelstaedt 1950). In this scheme, if actual sensory feedback is inconsistent with the predicted sensory outcomes, then the signals are attributed to an external, environmental source. In the case of a match between the predictions and actual sensory feedback, the cause of the signals is interpreted as being one's own action. This essentially results in enhancement of externally produced sensations and suppression of internally generated ones. This is reflected, for example, in the findings of Blakemore and colleagues (1999, 2000), who demonstrated a notable decrease in the “ticklishness” of a stimulus with decreasing spatial and temporal separation between self-produced and externally produced tactile stimulation. Similarly, perceptual steadiness, observed despite changes in retinal images during eye motion (Crowell et al. 1998; Lindner et al. 2001) or changes in body posture (Brooks and Cullen 2013; Cullen et al. 2011), is thought to be dependent on a similar suppression of self-produced sensations. Thus collectively, these studies suggest that predicted sensory outcomes of self-motion, when aligned with actual sensory feedback, can ultimately be used to attenuate sensory afference. Our current results, however, suggest that sensory predictions need not always to be used for suppressing the perception of sensations. Rather, they can be combined with sensory feedback to enhance perceptual estimates of the state of the body and the world; in our study, alignment of sensory feedback about target motion during the visual search task with the subjects' modified or natural default predictions always resulted in better perceptual decisions (when these predictions were stable). Our findings thus agree with newer studies that have suggested, for example, that sensory predictions generated as a consequence of saccadic eye movement can be used to provide a more reliable estimate of the position of the target of that movement (Vaziri et al. 2006). Our findings are also in line with the report of Phillips-Silver and Trainor (2005), who demonstrated enhanced auditory perceptual encoding of particular rhythm patterns in infants who were moved congruent with those patterns. Desantis et al. (2014) have recently proposed an elegant model to explain the divergent findings of suppression and enhancement of perceptual estimates that are derived using sensory predictions. They suggest that perception is attenuated when the intensity of a stimulus needs to be reported. In contrast, perception is enhanced when stimulus identification is required. Our task could be viewed as requiring stimulus identification, in that subjects had to identify and report the color of a moving target. This may then help to explain the enhanced accuracy of the color report when the target moved consistent with the subjects' predictions. Importantly, however, our study significantly expands the scope of prior studies by demonstrating that perceptual judgments appear to be influenced most strongly by the most stable predictions available. The stability in the predictive mechanism or forward model can be modified with training and cognitive load, as we have shown here, or conditions, such as aging, fatigue, or disease (Shadmehr et al. 2010), thus enslaving perceptual judgments to these conditions.

### Implications for Computational Models

Our results are aligned with the suggestions of computational models that propose that “priors” and sensory feedback are integrated in a Bayesian manner for optimal perception (Kording and Wolpert 2004; Kwon and Knill 2013; Stocker and Simoncelli 2006). Indeed, these priors can be viewed as predictions (Shadmehr et al. 2010), thus effectively equating the suggestions of the Bayesian models and our experiments here. It must be pointed out, however, that these computational models typically suggest that if the priors are unreliable, then perception is governed by sensory information alone. This is partly due to the fact that these models only include a single prior, in which unreliability is reflected as a large variance in the prior distribution. However, there is evidence that humans can learn more than one prior (Nagai et al. 2012), and it is not unrealistic to imagine that one of these priors could be unreliable. Our results indicate that in such cases, rather than relying on sensory information alone, as is generally suggested by Bayesian models, the nervous system adaptively transitions to using other stable priors for perceptual judgments. This is reflected in the fact that despite actual visual feedback about target motion being completely informative and reliable, subjects did not rely on it solely when the newly adapted prior was unreliable. If this were the case, then the accuracy would be similar, regardless of whether the target moved along the rotated path aligned with the hand or randomly. Instead, the results of our *experiment 2* suggest that subjects demonstrate an early reliance on their (weakly) adapted priors, but since these are fragile, they switch to using their natural priors, which were likely developed over their lifetime and were plausibly more stable. Even in *experiment 1*, the late reduction in accuracy of the color report of the target moving in the rotated direction and the slight (nonsignificant) but robust increase in accuracy for the hand-aligned target (bins 9 and 10; Fig. 3*B*) could be consequences of the operation of the same mechanism, albeit at a much longer time scale, since the artificially imposed predictions were stable for much longer. Our findings thus also have implications for computational models of perception and suggest that these models incorporate such flexible reliance on the most stable priors available when deriving perceptual estimates.

### Role of Attention and Eye Movements

It could be argued that attentional factors contribute to the greater accuracy in the visual search task under some conditions. A number of studies suggest that targets presented near or congruent with the visible or invisible hand are detected more rapidly (Reed et al. 2006, 2010), and people shift their attention away from such near-hand targets more slowly (Abrams et al. 2008). Such effects likely arise due to activation of bimodal sensory neurons in regions of the intraparietal sulcus, the supramarginal gyrus, and the dorsal and ventral premotor cortices (Graziano 1999; Graziano et al. 1994). Thus in the context of our results, it might appear that targets that move consistent with the subjects' predictions are perceptually more salient and are allocated greater attentional resources. This, in turn, allows them to be identified faster or more easily than other targets. However, it is important to recognize that there are two components to our visual search task: first, the target must be identified, and second, its color must be reported. Even if targets moving consistent with the subjects' predictions are indeed identified earlier, because they enjoy greater attentional priority, it is unclear how this translates into greater accuracy of the color report. Another possibility, however, is that accuracy was greater for these targets, because attentional priority to them allowed their features to be extracted within the allocated time. In contrast, there may simply not have been enough time to extract the required features of the target in the other conditions, since these conditions do not entail attentional priority, leading to poorer accuracy. However, subjects had enough time to search for and report the color of the target in all conditions; the subjects' RTs were much smaller than the maximum duration of a search trial. Furthermore, if time were not enough, we should have seen cases in which the target was simply not found. This was not the case either. Thus it appears that attentional factors did not specifically facilitate target identification. However, it is plausible that once a target was identified, attention was used to “lock in” on it so that if it moved consistent with the subjects' predictions, then tracking it among the moving distractors was less challenging compared with other target motion conditions (Steinbach and Held 1968; Vercher et al. 1995). This could allow subjects to spend more time with their eyes on the target, which could then enable the development of a better perceptual representation, ultimately enhancing the accuracy of the color report. However, if subjects spend more time on the target to allow a better representation to develop, then the time taken to report the color, manifested ultimately in the RT measure, could be longer. We did not observe this; RTs across the different target motion conditions were identical. One could still argue that a better representation could be developed within the same (reaction) time as the other target motion conditions, because tracking the target was easier. However, this raises another question of why, under the other target motion conditions, i.e., when the target did not move consistent with the predictions, subjects did not spend more time developing a better representation to increase their color report accuracy; more time was indeed available to do so. Future studies that integrate eye tracking with such paradigms can provide more insight into these issues.

### Potential Neural Substrates

Finally, it must be mentioned that previous research has suggested that the cerebellum is crucial for predicting the sensory consequences of action. Cerebellar lesions disrupt predictive control and also prevent recalibration of sensory predictions (Bastian 2006; Izawa et al. 2012; Synofzik et al. 2008). This suggests that in experiments, such as ours here, the advantage that sensory predictions provide for perceptual decisions should not be seen in individuals with cerebellar damage. More specifically, we expect that patients with cerebellar lesions will demonstrate similar accuracy across all target motion conditions in the perceptual task. This could be examined in future studies.

### Conclusion

In conclusion, we have shown that during active movement, the accuracy of decisions about perceptual attributes of moving stimuli is better if actual sensory feedback and sensory predictions are aligned and can be integrated. Importantly, in case these predictions are unstable or unreliable, the perceptual system appears to transition to using other stable predictions to enhance perceptual judgments rather than simply relying on sensory feedback alone. By uncovering this flexibility, our findings provide new insight into the organization of the perceptual system and also propose refinement of typical computational models of perception.

## GRANTS

Support for this work was provided by the Ramanujan Fellowship, Department of Science and Technology, Government of India (to P. K. Mutha), and the Wellcome Trust-DBT India Alliance Early Career Fellowship (to N. Kumar).

## DISCLOSURES

No conflicts of interest, financial or otherwise, are declared by the authors.

## AUTHOR CONTRIBUTIONS

Author contributions: N.K. and P.K.M. conception and design of research; N.K. performed experiments; N.K. and P.K.M. analyzed data; N.K. and P.K.M. interpreted results of experiments; N.K. and P.K.M. prepared figures; N.K. and P.K.M. drafted manuscript; N.K. and P.K.M. edited and revised manuscript; N.K. and P.K.M. approved final version of manuscript.
